# World-Wide Variation in Incidence of *Staphylococcus aureus* Associated Ventilator-Associated Pneumonia: A Meta-Regression

**DOI:** 10.3390/microorganisms6010018

**Published:** 2018-02-27

**Authors:** James C. Hurley

**Affiliations:** 1 Rural Health Academic Center, Melbourne Medical School, University of Melbourne, Ballarat, VIC 3350, Australia; jamesh@bhs.org.au; Tel.: +61-3-5320-4322; 2 Division of Internal Medicine, Ballarat Health Services, Ballarat, VIC 3350, Australia

**Keywords:** intensive care unit, geographic variation, ventilator-associated pneumonia, *Staphylococcus aureus*, MRSA

## Abstract

*Staphylococcus aureus* (*S. aureus*) is a common Ventilator-Associated Pneumonia (VAP) isolate. The objective here is to define the extent and possible reasons for geographic variation in the incidences of *S. aureus*-associated VAP, MRSA-VAP and overall VAP. A meta-regression model of *S. aureus*-associated VAP incidence per 1000 Mechanical Ventilation Days (MVD) was undertaken using random effects methods among publications obtained from a search of the English language literature. This model incorporated group level factors such as admission to a trauma ICU, year of publication and use of bronchoscopic sampling towards VAP diagnosis. The search identified 133 publications from seven worldwide regions published over three decades. The summary *S. aureus*-associated VAP incidence was 4.5 (3.9–5.3) per 1000 MVD. The highest *S. aureus*-associated VAP incidence is amongst reports from the Mediterranean (mean; 95% confidence interval; 6.1; 4.1–8.5) versus that from Asian ICUs (2.1; 1.5–3.0). The incidence of *S. aureus*-associated VAP varies by up to three-fold (for the lowest versus highest incidence) among seven geographic regions worldwide, whereas the incidence of VAP varies by less than two-fold. Admission to a trauma unit is the most important group level correlate for *S. aureus*-associated VAP.

## 1. Introduction

Ventilator-Associated Pneumonia (VAP) in association with *Staphylococcus aureus* has been reported from over 100 intensive care units (ICU) worldwide [1,2,3,4,5,6,7,8,9,10,11,12,13,14,15,16,17,18,19,20,21,22,23,24,25,26,27,28,29,30,31,32,33,34,35,36,37,38,39,40,41,42,43,44,45,46,47,48,49,50,51,52,53,54,55,56,57,58,59,60,61,62,63,64,65,66,67,68,69,70,71,72,73,74,75,76,77,78,79,80,81,82,83,84,85,86,87,88,89,90,91,92,93,94,95,96,97,98,99,100,101,102,103,104,105,106,107,108,109,110,111,112,113,114,115,116,117,118,119,120,121,122,123,124,125,126,127,128,129,130,131,132,133]. In two series drawn predominantly from ICUs in The United States of America and Europe, *S. aureus* accounted for 20% [134] and 22% [135] of bronchoscopically-documented cases of VAP. 

Whether VAP is associated with an increase in attributable mortality may depend on the infecting organism [8,12,115,135]. There may be a specific mortality risk for ventilator-associated pneumonia in association with *S. aureus* infections in the ICU, although this may be influenced by associated resistance to methicillin [136,137]. 

There is a worldwide variation in the microbial aetiologies of VAP and other ICU-acquired infections [1,2,3,4,5,6,8,115,130,131,132,133,134,135,136,137,138,139,140]. For example, the incidence of *Acinetobacter*-associated VAP varies five-fold among reports from ICUs from various geographic regions around the world [139]. 

The objective here is to define the extent of geographic variation in the incidence of VAP associated with *S. aureus* within the published literature versus the variation in the incidences associated with VAP overall and with MRSA-VAP. An additional aim is to explore the degree to which any variation may be explainable by other study-related factors, such as mode of VAP diagnosis or admission for trauma [67], using meta-regression methods. 

## 2. Methods

Because this analysis was based on a literature survey, institutional review board approval was not required.

The literature search and analytic approach used here has been adapted from one used previously [139]. In brief, an electronic search of PubMed, the Cochrane database and Google Scholar for systematic reviews containing potentially eligible studies was done using the following search terms: “ventilator-associated pneumonia”, “mechanical ventilation”, “intensive care unit”. The study inclusion criteria were as follows; a listing of *S. aureus* among the VAP isolates, reporting in the English language and reports for which a VAP incidence could be estimated using the number of Mechanical Ventilation Days (MVD) as the denominator. A hand search was undertaken for additional studies not identified within systematic reviews.

Studies that were restricted to eligible patients within randomized controlled trials were not included as these generally limit inclusion to patients meeting specific eligibility criteria. However, studies that were undertaken without patient restriction within the context of process improvement for the purpose of general infection prevention were retained in the model and designated ‘intervention period’ studies. Studies that were limited to paediatric, burns [141] or haemato-oncology ICUs were excluded.

The *S. aureus*-associated VAP incidence is defined as the number of patients with VAP having *S. aureus* isolated from respiratory sampling per 1000 MVD. Where necessary, the numerator was derived as the number of patients with VAP multiplied by the proportion of VAP isolates that were *S. aureus*. This approximation allows for VAP patients with multiple isolates. In addition, the following were also extracted where available: the number of ICU patients surveyed, the overall incidence of VAP per 1000 mechanically ventilated days, whether the mode of diagnosis of VAP required bronchoscopic sampling and whether the ICU was a trauma ICU (defined as more than 50% of patient admissions being for trauma). 

The assignment of countries to near neighbour groupings was solely determined in relation to geographic proximity without regard to political, economic or other considerations. It was not always clear as to the dates to which each survey applied. For convenience and for uniformity, the year of publication rather than the year of the study has been used as a covariate in the meta-regression models and the figures. Meta-regression models of VAP overall, *S. aureus*-associated VAP and MRSA-VAP were undertaken using the relevant inverse of the variance for each as the study weighting. Because heterogeneity is to be expected both within and between regions, a random effects method was used in deriving summary estimates. The following predictor variables were used without pre-selection in the regression model: the geographic region, whether bronchoscopic sampling was used in the diagnosis of VAP, trauma ICU, year of publication and whether an infection prevention intervention was in place. All factors were entered into the regression models without any pre-selection step. For the purpose of the meta-regression models, the groups from multinational studies and those from studies that were ungrouped were collapsed into a single group, and this composite group was used as the reference group.

### Availability of Data and Materials

The datasets supporting the conclusions of this article are included within the article and its additional file.

## 3. Results

**Figure 1:**
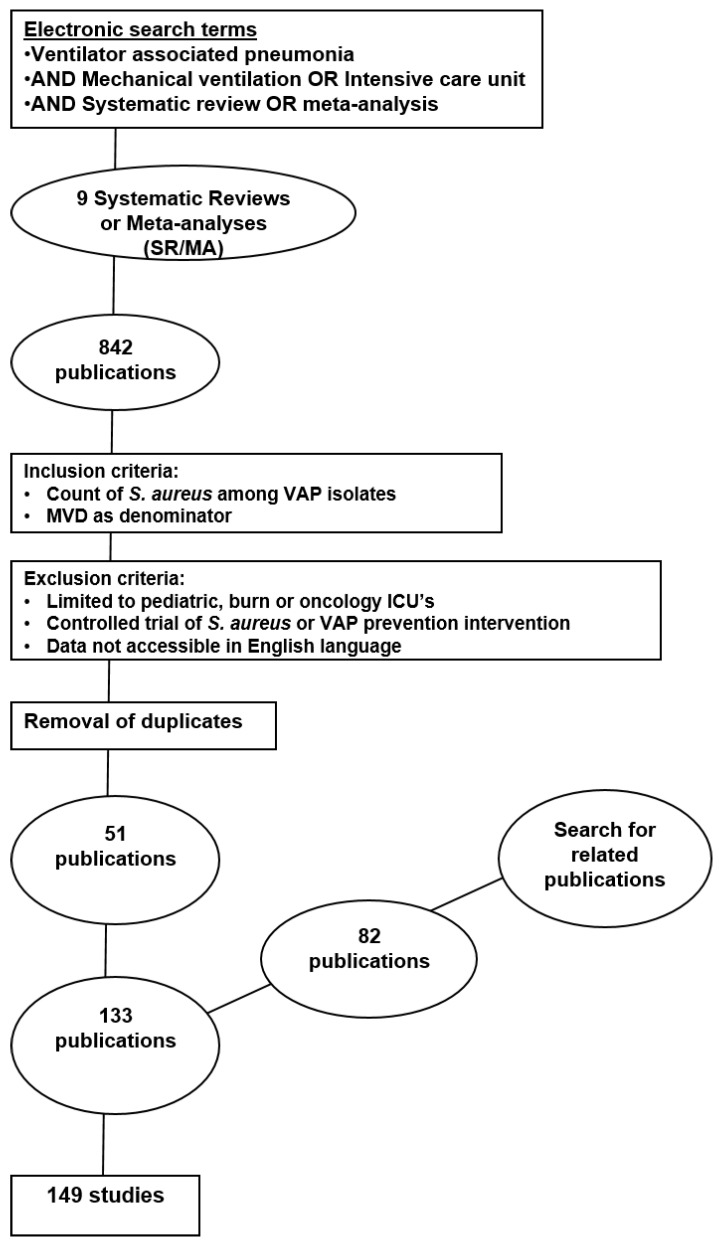
Flowchart of the literature search. MVD, Mechanical Ventilation Days; VAP, Ventilator-Associated Pneumonia; SR/MA, systematic review or meta-analysis.

The search identified 149 study groups contained in 133 publications published between 1986 and 2018 [1,2,3,4,5,6,7,8,9,10,11,12,13,14,15,16,17,18,19,20,21,22,23,24,25,26,27,28,29,30,31,32,33,34,35,36,37,38,39,40,41,42,43,44,45,46,47,48,49,50,51,52,53,54,55,56,57,58,59,60,61,62,63,64,65,66,67,68,69,70,71,72,73,74,75,76,77,78,79,80,81,82,83,84,85,86,87,88,89,90,91,92,93,94,95,96,97,98,99,100,101,102,103,104,105,106,107,108,109,110,111,112,113,114,115,116,117,118,119,120,121,122,123,124,125,126,127,128,129,130,131,132,133]. Of the 133 publications found, 51 had and 82 had not been cited within one of eight systematic reviews identified by the search [142,143,144,145,146,147,148,149] (Figure 1). Fifteen publications provided more than one study. The studies are detailed in Table S1 (see the additional file). The studies were classified by geographic region as detailed in Table 1. There were 17 multinational ICU surveys from six publications that were derived from ICUs that had been anonymized by originating country in these publications.

There were 21 studies that reported for trauma ICU populations. While none of the studies were undertaken in an ICU subject to a known outbreak, there were nine studies undertaken in the context of an infection control intervention. The use of bronchoscopic sampling in the diagnosis of VAP was unequal among the seven regions being used in more than half of the studies from Northern Europe and the Mediterranean versus less than half of studies elsewhere (*p* < 0.001; chi-square = 27.13, 6 df).

**Figure 2:**
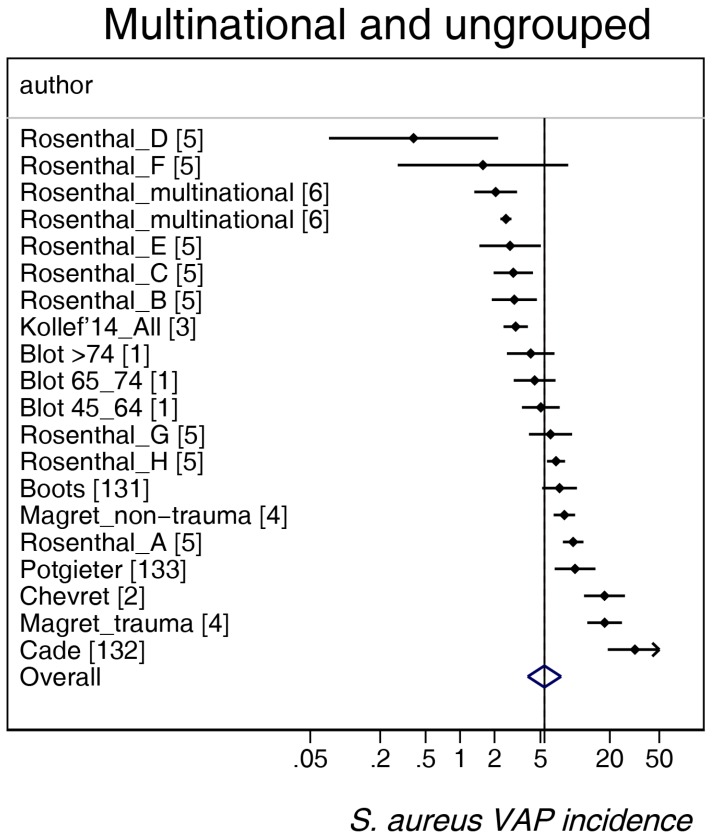
Caterpillar plots of the group-specific (small diamonds) and summary (large open diamond, vertical line) *S. aureus* VAP incidence per 1000 mechanical ventilation days and 95% CI for groups from the multinational and ungrouped studies. Studies are listed in Table S1 (see the additional file). Note that the *x* axis is a logarithmic scale.

**Figure 3:**
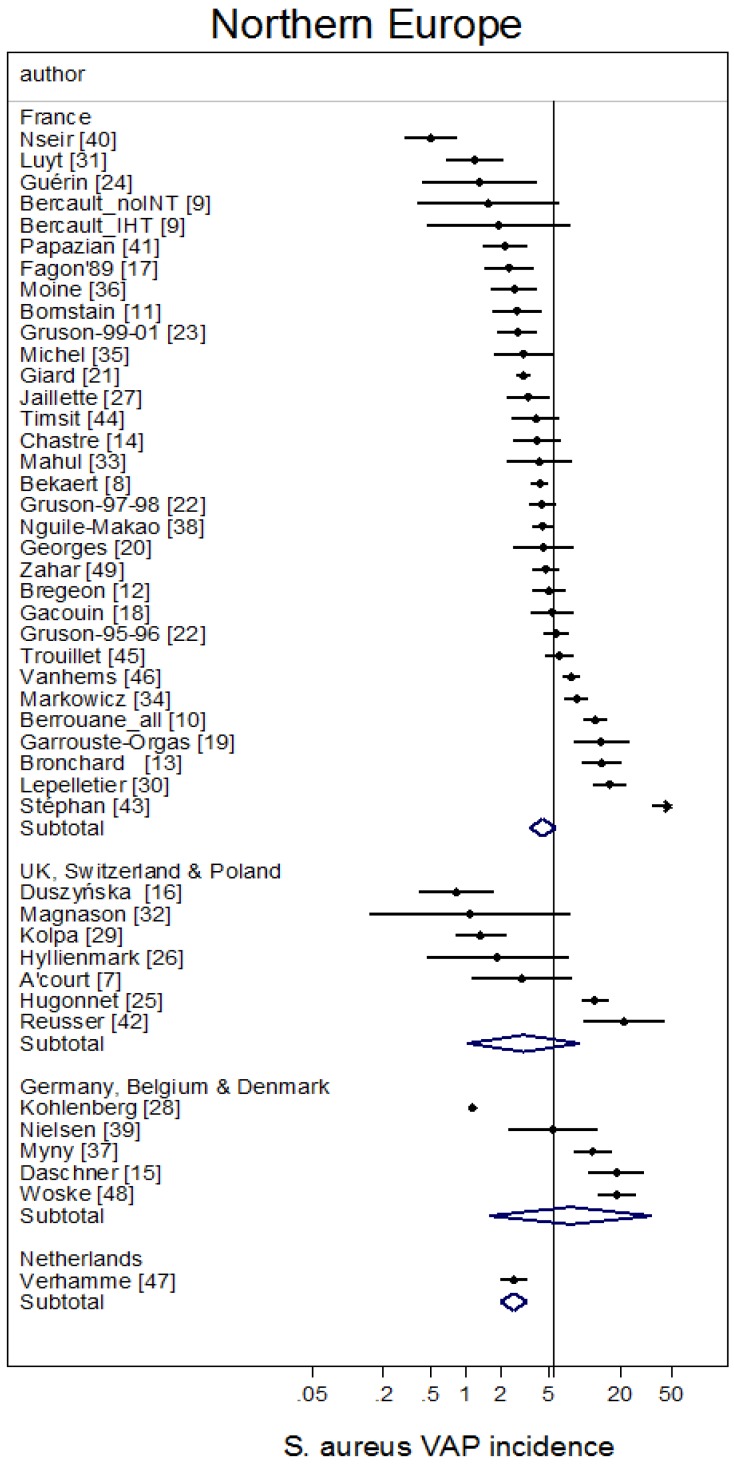
Caterpillar plots of the group-specific (small diamonds) and summary (large open diamond) *S. aureus* VAP incidence per 1000 mechanical ventilation days and 95% CI for groups from Northern European countries. For comparison, the summary *S. aureus* VAP incidence (vertical line) derived from the studies in Figure 2 is shown for reference. Studies are listed in Table S1 (see the additional file). Note that the *x* axis is a logarithmic scale.

**Figure 4:**
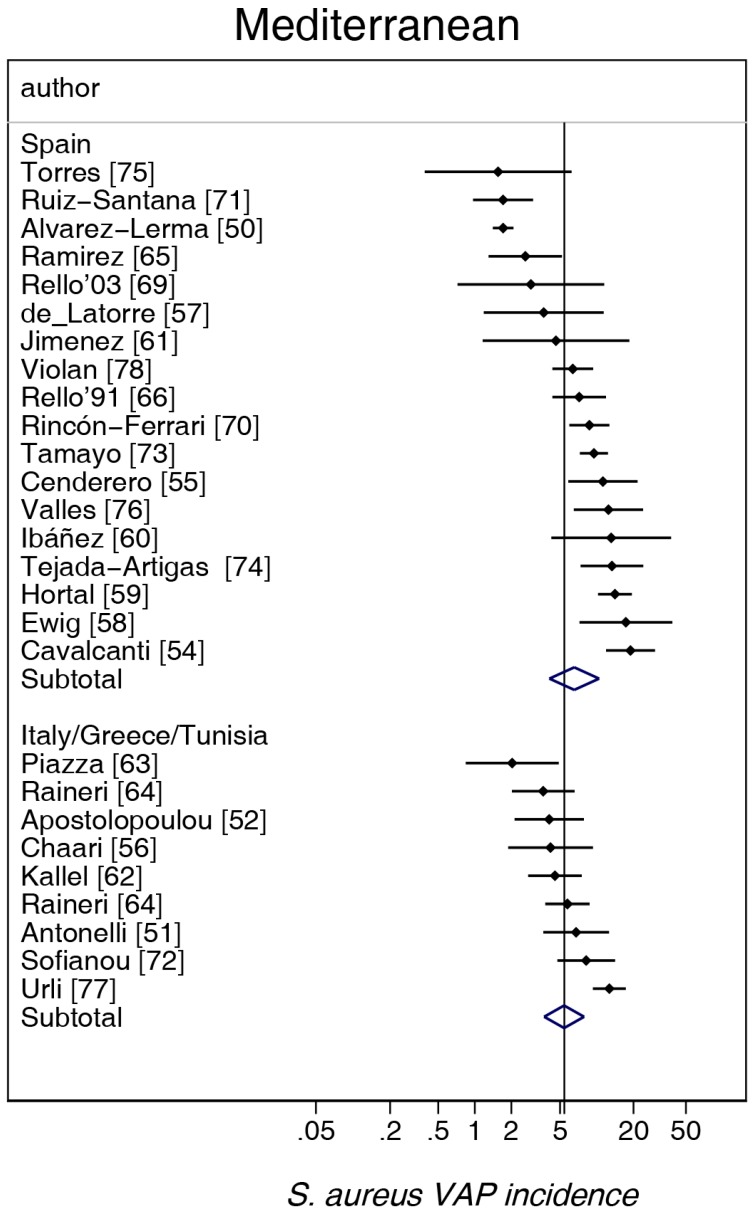
Caterpillar plots of the group-specific (small diamonds) and summary (large open diamond) *S. aureus* VAP incidence per 1000 mechanical ventilation days and 95% CI for groups from the Mediterranean studies. For comparison, the summary *S. aureus* VAP incidence (vertical line) derived from the studies in Figure 2 is shown for reference. Studies are listed in Table S1 (see the additional file). Note that the *x* axis is a logarithmic scale.

**Figure 5:**
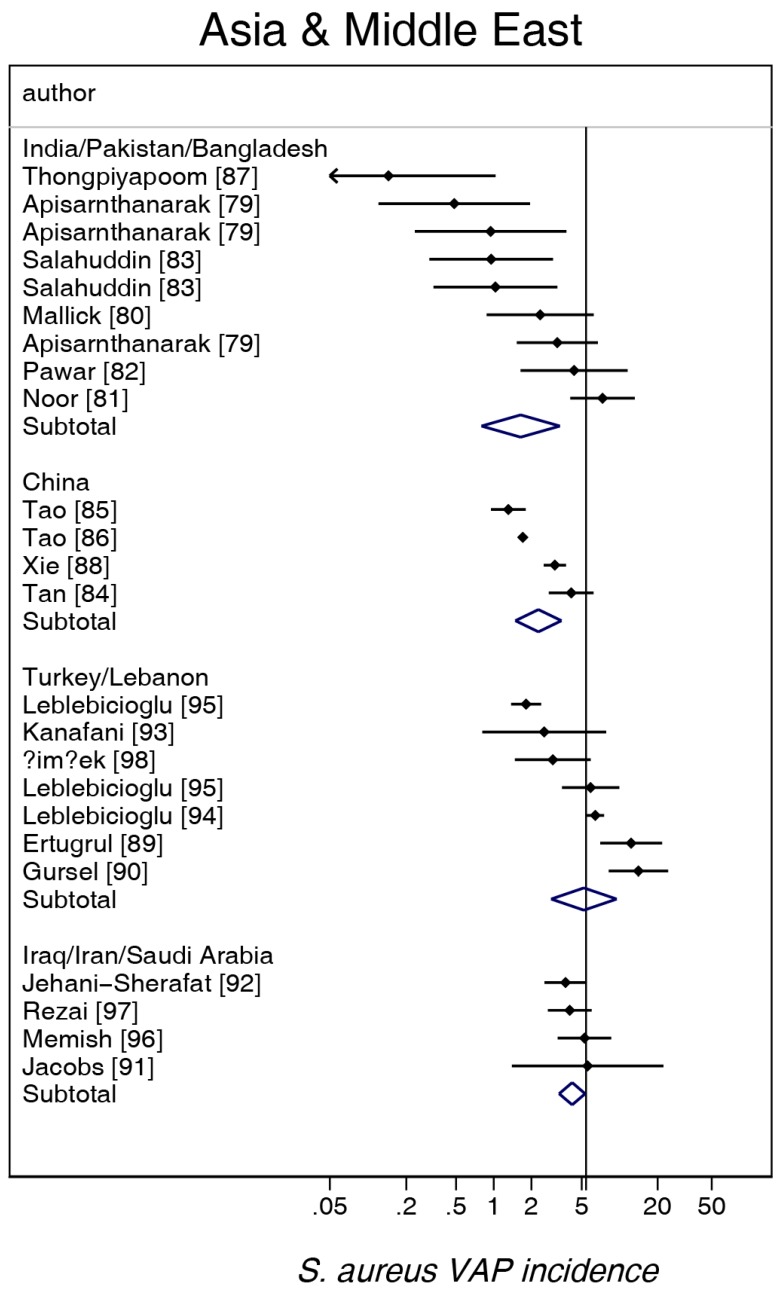
Caterpillar plots of the group-specific (small diamonds) and summary (large open diamond) *S. aureus* VAP incidence per 1000 mechanical ventilation days and 95% CI for groups from the studies from Asia and the Middle East. For comparison, the summary *S. aureus* VAP incidence (vertical line) derived from the studies in Figure 2 is shown for reference. Studies are listed in Table S1 (see the additional file). Note that the *x* axis is a logarithmic scale.

**Figure 6:**
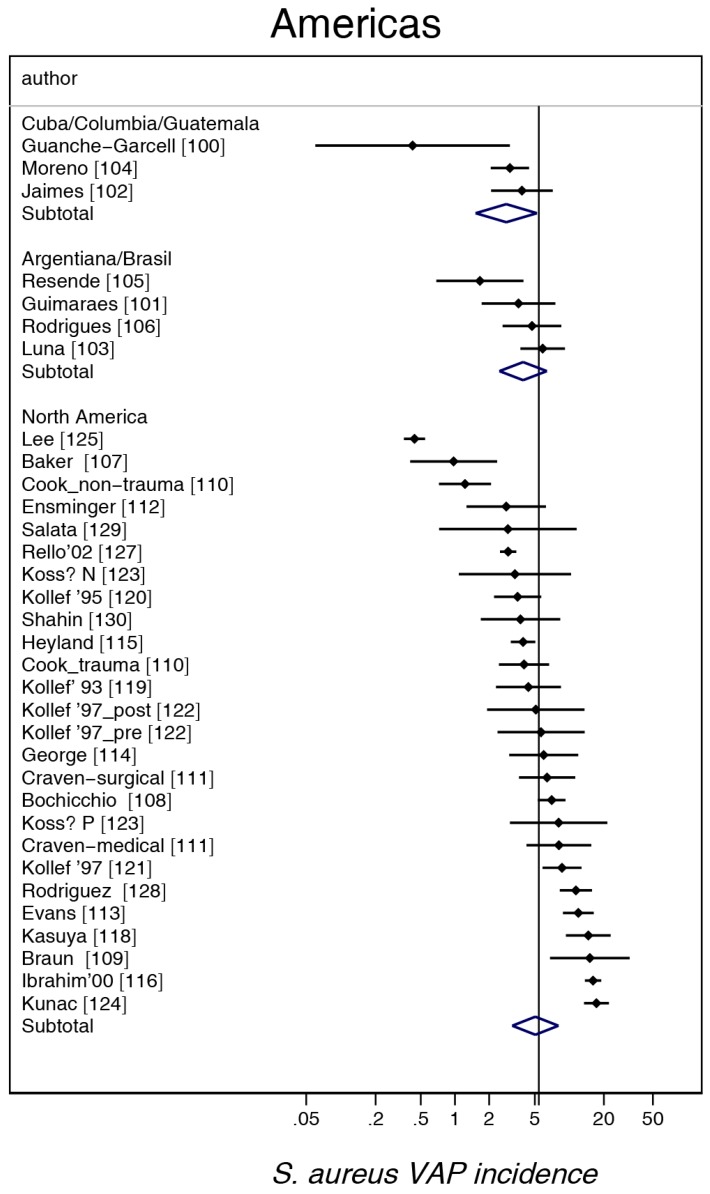
Caterpillar plots of the group-specific (small diamonds) and summary (large open diamond) *S aureus* VAP incidence per 1000 mechanical ventilation days and 95% CI for groups from the North and Central and South American studies. For comparison, the summary *S. aureus* VAP incidence (vertical line) derived from the studies in Figure 2 is shown for reference. Studies are listed in Table S1 (see the additional file). Note that the *x* axis is a logarithmic scale.

**Figure 7:**
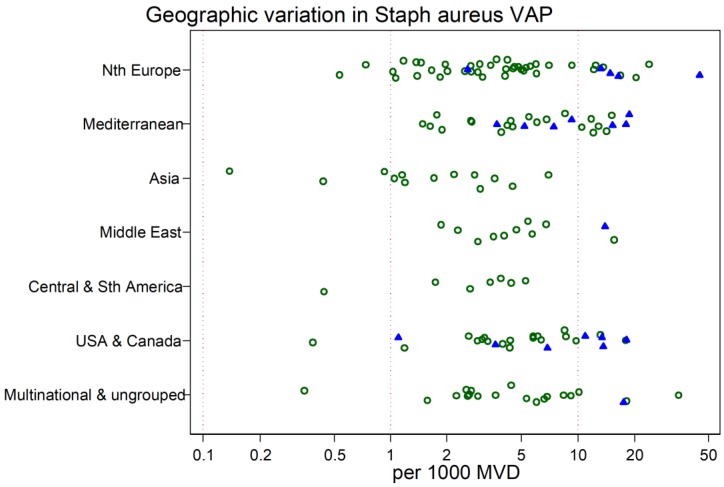
Scatter plot of *S. aureus* VAP incidence (per 1000 MV days) among published studies in seven geographic regions with rates for studies reporting from trauma ICUs (closed symbols) vs. other ICUs (open symbols). Note the logarithmic scale of incidence. The vertical lines are for reference at incidence rates of 0.1, 1 and 10 per 1000 MV days.

**Figure 8:**
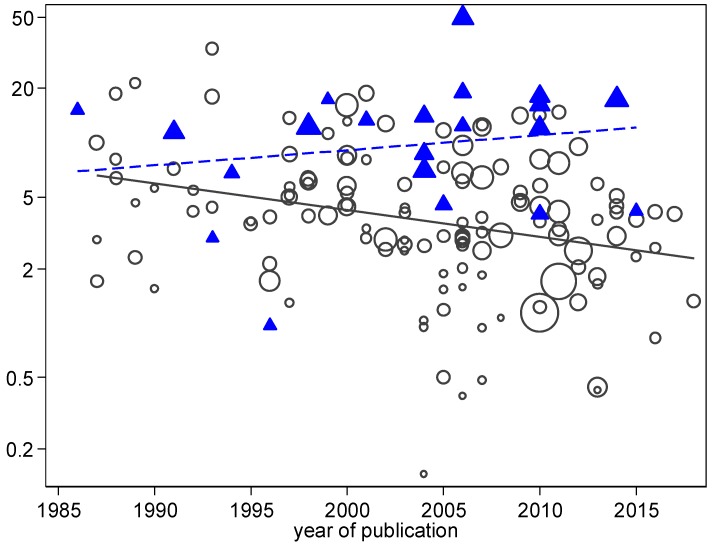
Scatter plot and linear regression of *S. aureus* VAP incidence (per 1000 MV days) versus year of study publication for studies reporting from trauma ICUs (triangles, broken line) versus non-trauma ICUs (circles, unbroken line). Note the logarithmic scale of incidence. The slopes of the linear regression lines are significantly different (test for interaction *p* < 0.001; Poisson regression).

**Figure 9:**
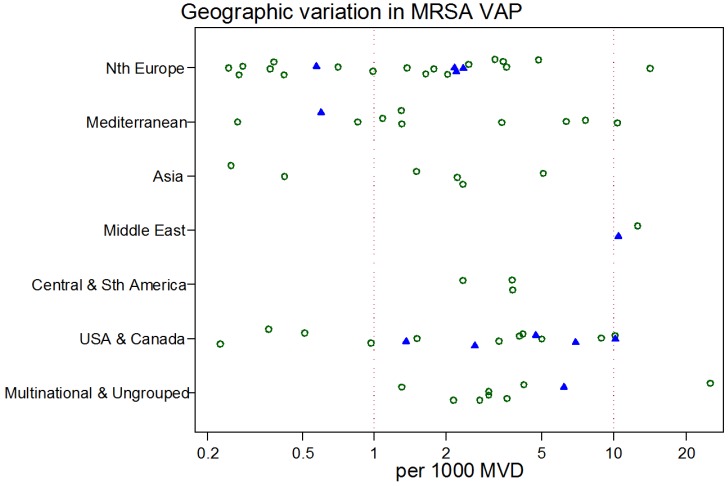
Scatter plot of MRSA-VAP incidence (per 1000 MV days) among published studies in seven geographic regions with rates for studies reporting from trauma ICUs (closed symbols) versus other ICUs (open symbols). Note the logarithmic scale of incidence. The vertical lines at incidences of 1 and 10 per 1000 MVD are for reference.

The study-specific *S. aureus*-associated VAP incidence is displayed by regions (Figure 2, Figure 3, Figure 4, Figure 5 and Figure 6) and collectively with all studies together (Figure 7 and Figure 8). The incidence of MRSA-VAP are displayed by year of publication (Figure S1) and collectively by country (Figure 9). Over all 162 groups, the summary incidence of VAP was 21.3 (18.9–23.8) per 1000 MVD (Figure S2) and 17.1 (14.0–20.6) per 100 patients. The summary *S. aureus*-associated VAP incidence was 4.5 (3.9–5.3) per 1000 MVD and 3.4 (2.6–4.5) per 100 patients. The numbers of MRSA VAPs were reported for 55 studies, and the summary was 2.2 (1.6–3.1) per 1000 MVD.

The highest and lowest *S. aureus*-associated VAP incidences were amongst reports from Mediterranean versus Asian ICUs, respectively. By contrast, the highest and lowest incidences of VAP overall were amongst reports from Mediterranean versus Northern European ICUs, respectively. The incidence of MRSA VAP was lowest within reports from Northern European ICUs (Table 1).

Meta-regression models of VAP incidence, *S. aureus*-associated VAP and MRSA-VAP incidence are presented in Table 2. In the meta-regression model for VAP incidence overall and for *S. aureus*-associated VAP incidence, origins from a trauma ICU, but not mode of diagnosis using bronchoscopic sampling were significant correlates. In these models, origin from a Northern European study was significantly associated with lower incidences of VAP overall and MRSA-VAP, but not *S. aureus* VAP. In none of the three models was the year of publication significantly associated with incidence. However, a closer examination revealed an interaction in that a decline in *S. aureus*-associated VAP (Figure 8) and MRSA-VAP (see the additional file: Figure S2) incidences over the years was apparent for reports from non-trauma ICUs, but not for trauma ICUs (*p* = 0.001).

## 4. Discussion

This is a survey of the incidences of VAP overall, *S. aureus*-associated VAP and MRSA-VAP among over 100 published studies using meta-analysis. The incidences of *S. aureus*-associated VAP and MRSA-VAP each vary by up to three-fold for the lowest versus highest incidence region among seven geographic regions worldwide, whereas the incidence of VAP varies by less than two-fold. 

This variation in incidence among seven broad multinational regions is partly explainable by a limited number of group level factors. Of note, in the meta-regression models, trauma is more strongly correlated with *S. aureus*-associated VAP than was the case for the incidence of VAP overall or MRSA-VAP. A decline in the incidences of *S. aureus*-associated VAP and MRSA-VAP in association with year of publication is apparent only for reports originating from non-trauma ICUs. 

The less than three-fold variation in *S. aureus*-associated VAP contrasts with the greater than five-fold variation in *Acinetobacter*-associated VAP incidence observed in an analysis of mostly the same studies as included here [139].

The findings here reinforce and further characterize previous observations in four multi-national surveys [1,3,5,140]. Rello et al. surveyed ICUs in the following four regions: Paris, Barcelona, Montevideo and Seville, and likewise found less variation between the sites in *S. aureus*-associated VAP than was the case for *Acinetobacter*-associated VAP [141]. Another multi-national prospective 24-month survey [5] was conducted across 55 ICUs of 46 hospitals in Argentina, Brazil, Colombia, India, Mexico, Morocco, Peru and Turkey. This anonymized survey also found less variation in *S. aureus*-associated VAP than was the case for *Acinetobacter*-associated VAP. This survey found an overall rate of VAP of 24.1 per 1000 MV days with *S. aureus* accounting for between 13 and 53% of VAP isolates amongst the eight anonymized countries [5]. 

Kollef et al. prospectively surveyed VAP among 1873 mechanically-ventilated patients in 56 ICUs from 11 countries in the following four regions: Europe, United States, Latin America and the Asia-pacific region [3]. This survey [3] found that the incidence per 100 patients of VAP overall, *S. aureus* VAP and MRSA-VAP among all 56 ICUs was 293/1873 (15.6%), 65/1873 (3.5%) and 27/1873 (1.4%), respectively. This compares to incidence proportions for VAP overall and *S. aureus* VAP found here being 17.1 (14.0–20.6), 3.4 (2.6–4.5) and 1.95 (1.14–3.3), respectively. Interestingly, these investigators found that the incidence of VAP overall, but not the incidence of *P. aeruginosa* VAP varied among the four broadly-defined worldwide regions. In this study [3], the incidences of *S. aureus* VAP and MRSA-VAP were each only reported in aggregate.

Finally, a survey of 27 European ICUs found a summary VAP incidence of 18.3 per 1000 MVD and that the dominant nosocomial pneumonia isolate varied among the nine European countries in the survey [1,138]. *S. aureus* was the dominant pneumonia isolate in Spain, France, Belgium and Ireland [1,138]. 

The extent to which any possible variation in VAP microbiology between regions is explainable by group level factors is difficult to establish in studies that are either short term or single centre. However, the findings here that admissions for trauma, but not bronchoscopic sampling undertaken as part of VAP diagnosis are significant factors towards *S. aureus* VAP are in line with findings reported from single centre studies [150,151].

With a literature survey, a considerable convenience is that the data are readily available, and the meta-regression methods for analysing these types of data are established. A random effects meta-regression methods is used as previously to enable the imprecision associated with each individual study estimate to be incorporated in the derivation of both the summary estimates and in the meta-regression models [139,152]. This is the major advantage of a meta-analysis in comparison to a simple narrative review. Estimates with random effects are more conservative (i.e., wider 95% confidence limits) than with fixed effects. This analytic approach enables an appreciation of the contextual influences of study-specific factors that would not be apparent within a single centre study examined in isolation. 

There are several limitations to this analysis of the literature. Of particular note, this is not a systematic review, and the analysis is limited to English language articles. This is an analysis at the group level and therefore is unable to take account of patient-specific risk factors for *S. aureus*-associated VAP. For example, the duration of mechanical ventilation and usage of empiric antibiotic therapy are important VAP determinants at the level of the individual patient that cannot be explored at the group level of analysis. To include patient level information within a meta-analysis would require an individual patient data meta-analysis. 

A further limitation relating to the survey of MRSA-VAP is that in some reports, MRSA-VAP may not have been reported as a consequence of being rare in that specific location. Hence, this reporting bias may lead to under-representation of zero counts. Moreover, the analysis is unable to account for strain type differences underlying the variation in both *S. aureus* VAP incidence and also in MRSA-VAP incidence. The ‘intervention periods’ studies include a broad range of interventions. Their inclusion is to indicate the influence relative to other group level associates on the incidences. However, the methodology used here is not appropriate for the evaluation of the effectiveness of infection control interventions. Randomised controlled trials were not included here for two reasons. Firstly, the populations of these studies usually are a small sub-group that meets specific inclusion criteria. Moreover, there is the potential for a contextual effect in the presence of any such intervention within an ICU on the incidence of VAP overall [152], and more so in relation to the potential contextual effect of prevention interventions on *S. aureus*-associated VAP [151,152,153].

As with any multi-national survey, a range of definitions was applied in the diagnosis of VAP among the studies here, and standardisation was not possible. The classification of studies into those that did versus did not use bronchoscopic sampling towards VAP diagnosis was a simplifying compromise. 

The grouping of countries into near neighbour groupings is somewhat arbitrary. This grouping may conceal important variations within each of the regions. Country and even regional groupings could be confounded by other variables such as infection control practices, prevalence of antibiotic use and standards of care for patients receiving mechanical ventilation, which are not able to be considered in the analysis here. Indeed, even the imperative to publish may differ in different countries. The influence of publication bias is difficult to estimate.

## 5. Conclusions

There is a variation of up to three-fold in *S. aureus*-associated VAP and MRSA-VAP among published reports from seven broad geographic regions worldwide. This variation exceeds the variation in incidence of VAP overall. For MRSA-VAP incidence, there is a complex interaction between the year of publication and admission for trauma.

## Figures and Tables

**Table 1:** Characteristics of the studies ^a^.

	Multinational and Ungrouped	Northern Europe ^b^	Mediterranean ^c^	Asia ^d^	Middle East ^e^	Central and South America ^f^	USA/Canada ^g^
Sources [ref]	[1,2,3,4,5,6,131,132,133]	[7,8,9,10,11,12,13,14,15,16,17,18,19,20,21,22,23,24,25,26,27,28,29,30,31,32,33,34,35,36,37,38,39,40,41,42,43,44,45,46,47,48,49]	[50,51,52,53,54,55,56,57,58,59,60,61,62,63,64,65,66,67,68,69,70,71,72,73,74,75,76,77,78]	[79,80,81,82,83,84,85,86,87,88]	[89,90,91,92,93,94,95,96,97,98]	[99,100,101,102,103,104,105,106]	[107,108,109,110,111,112,113,114,115,116,117,118,119,120,121,122,123,124,125,126,127,128,129,130]
Number of groups	20	45	27	13	11	7	26
Trauma ICUs ^h^	1	5	7	0	1	0	7
Bronchoscopic sampling ^i^	2	27	16	0	2	1	10
Intervention period ^j^	1	2	1	4	1	1	2
Study publication year (range)	1987–2014	1988–2018	1987–2016	2003–2016	1990–2017	2003–2013	1986–2014
Numbers of patients per study group; median (IQR)	1194; 411–2339	439; 175–1004	184; 101–318	618; 344–1076	448; 92–2584	274; 180–712	327; 223–521
Duration of MV (days); median (IQR)	7.4; 5–9.2	10.7; 8.0–13	8.0; 7–11	6.0; 2.5–9	9.8; 8.9–13.5	9.6; 7.6–10	6.0; 5–8
VAP incidence per 1000 MV days							
mean	25.0	17.8	26.8	18.2	24.0	21.8	20.5
95% CI	20.7–30.2	14.0–22.4	20.9–34.1	14.3–23.1	18.2–31.2	13.6–34.8	14.0–30.6
*S. aureus* VAP incidence per 1000 MV days							
mean	5.4	4.4	6.1	2.1	4.9	3.5	5.1
95% CI	3.9–7.5	3.2–6.1	4.4–8.5	1.5–3.0	3.3–7.3	2.4–5.0	3.2–8.0
*MRSA* VAP incidence per 1000 MV days ^k^							
mean	3.7	1.4	2.3	1.8			2.5
95% CI	2.2–6.1	0.8–2.5	1.1–4.5	0.9–3.3			1.2–5.3
*n*	9	22	10	6	2	3	16

^a^ Abbreviations; ICU, Intensive Care Unit; MV, Mechanical Ventilation; NA, Not Applicable; VAP, Ventilator-Associated Pneumonia; IQR, Interquartile Range; ^b^ Northern Europe includes France, Germany, the United Kingdom, Switzerland, Sweden, Iceland and Poland; ^c^ Mediterranean includes Spain, Italy, Greece and Tunisia; ^d^ Asia includes China, India, Pakistan and Bangladesh; ^e^ Middle East includes Turkey, Iraq, Lebanon and Saudi Arabia; ^f^ Central and South America includes Argentina, Brazil, Chile, Colombia, Cuba and Guatemala; ^g^ Northern America includes USA and Canada; ^h^ Trauma ICU defined as an ICU with >50% of patient admissions for trauma; ^i^ bronchoscopic vs. tracheal sampling toward the diagnosis of VAP; ^j^ number of groups that were studied during a period of an infection control intervention; ^k^ summary MRSA VAP incidences are not reported for regions with fewer than four reports.

**Table 2:** Log VAP incidence per thousand MV days; meta-regression models ^a^.

	Overall VAP			*S. aureus* VAP			MRSA VAP		
Factor	Coefficient ^b^	95% CI	*p*	Coefficient ^b^	95% CI	*p*	Coefficient ^b^	95% CI	*p*
Multinational and Ungrouped (reference group)	+3.60	+3.12–+4.08		+2.16	+1.54–+2.79		+1.99	+0.68–+3.31	
Geographic region									
Northern Europe	−0.37	−0.73–−0.01	0.05	−0.27	−0.74–+0.20	0.26	−1.09	−1.97–−0.21	0.02
Mediterranean	−0.06	−0.45–+0.34	0.78	−0.12	−0.63–+0.39	0.64	−0.69	−1.71–+0.33	0.18
Asia	−0.23	−0.69–+0.22	0.32	−0.79	−1.4–−0.18	0.01	−0.75	−1.95–+0.45	0.21
Middle East	−0.04	−0.51–+0.43	0.87	−0.04	−0.65–+0.56	0.88	+0.95	−0.66–+2.56	0.24
Central and South America	−0.08	−0.61–+0.44	0.76	−0.45	−1.17–+0.28	0.23	−0.22	−1.60–+1.16	0.75
USA and Canada	−0.35	−0.75–+0.05	0.08	−0.33	−0.83–+0.18	0.20	−0.62	−1.53–+0.29	0.18
Trauma ^c^	+0.38	+0.07–+0.68	0.02	+0.82	+0.43–+1.21	0.001	+0.29	−0.43–+1.00	0.42
Year of publication ^d^	−0.01	−0.03–+0.01	0.065	−0.02	−0.04–−0.01	0.04	−0.02	−0.06–+0.02	0.26
Mode of diagnosis ^e^	−0.07	−0.31–+0.16	0.53	+0.01	−0.30–+0.31	0.95	+0.13	−0.44–+0.70	0.65
Intervention period ^f^	−0.35	−0.74–+0.04	0.075	−0.50	−1.03–+0.04	0.068	−0.54	−1.67–+1.58	0.34

^a^ This table displays the results of meta-regression analyses for log VAP, log *S. aureus*-associated VAP and log MRSA VAP incidence per thousand MV days. ^b^ Interpretation. The reference group is the composite group of multinational and ungrouped studies, and this coefficient equals the difference in log from 0 (a log equal to 0 equates to a rate of 1). The other coefficients represent the difference in log for groups positive for that factor vs. the reference group. ^c^ The coefficient for trauma represents the increment in log for an ICU having a majority of admissions for trauma. ^d^ The coefficient for year of publication represents the linear increment in log for each year after 1980. ^e^ For sampling using bronchoscopic versus tracheal sampling. ^f^ Studies undertaken during an infection control intervention.

## Supplementary Materials

The following are available online at http://www.mdpi.com/2076-2607/6/1/18/s1. Table S1: Listing of studies reporting *S aureus* VAP & MRSA-VAP. Figure S1: MRSA-VAP incidence (per 1000 MV days) versus year of study publication. Figure S2: A scatter plot of VAP overall incidence worldwide.

Supplementary File 1
