# Supplementary material for: World-Wide Variation in Incidence of Staphylococcus aureus Associated Ventilator-Associated Pneumonia: A Meta-Regression
Source: Microorganisms. 2018 Feb 27;6(1):18. doi: 10.3390/microorganisms6010018 (PMC5874632; doi:10.3390/microorganisms6010018)
Supplement: Supplementary File 1 [file microorganisms-06-00018-s001.pdf]

Appendix: Table of contents

Table S1: Listing of studies reporting *S aureus* VAP & MRSA-VAP 2 - 8

References (s1-s133) 9 – 18

Fig S1 MRSA-VAP incidence (per 1000 MV days) versus year of study publication 19

Fig S2 A scatter plot of VAP overall incidence worldwide 20

Abbreviations for additional file

ICU, Intensive care unit;

MV; Mechanical ventilation

MVdays; Mean number of days of mechanical ventilation

MRSA; Methicillin resistant *Staphylococcus aureus*

VAP; Ventilator associated pneumonia

Table S1: Studies reporting *S aureus* VAP incidence

Author	Year	Ref	Notes[a]	MV (days) [b]	Patients (n)	VAP (n)	*S aureus* VAP (n)	MRSA VAP (n)
**Multinational**								
Blot 45-64 [d]	2014	1			670	103	27	15
Blot 65-74 [d]	2014	1			549	104	22	14
Blot >74 [d]	2014	1			516	73	17	9
Chevret	1993	2	B	1275	255	55	23	
Kollef	2014	3		21352	1873	293	65	27
Magret_non-trauma [d]	2010	4		10410	2082	337	84	42
Magret_trauma [d]	2010	4	T	1770	354	128	32	10
Rosenthal_C	2006	5		8593	2172	86	25	
Rosenthal_G	2006	5		3364	1359	98	21	
Rosenthal_H	2006	5		17222	2305	490	118	
Rosenthal_E	2006	5		3754	1514	73	10	
Rosenthal_F	2006	5		835	410	44	1	
Rosenthal_A	2006	5		9442	8867	284	91	
Rosenthal_D	2006	5		3401	3413	67	1	
Rosenthal_B	2006	5		6376	1029	135	19	
Rosenthal	2012	6		10292	3889	226	21	
Rosenthal	2012	6	I	127347	51618	2191	319	

Table S1: Studies reporting *S aureus* VAP incidence (continued)

Author	Year	Ref	Notes[a]	MV (days) [b]	Patients (n)	VAP (n)	*S aureus* VAP (n)	MRSA VAP (n)
**North Europe**								
A'court	1993	7		1350	150	33	4	
Bekaert	2011	8	B	31985	4479	685	133	49
Bercault_IHT	2005	9		1062	118	31	2	
Bercault_noINT	2005	9	I	1298	118	12	2	
Berrouane_all	2001	10	B	5796	565	129	71	14
Bornstain	2004	11	B	6349	747	80	17	6
Bregeon	1997	12	B	6798	660	223	34	
Bronchard	2004	13	B	1853	109	45	26	1
Chastre	1998	14	B	4568	769	268	18	
Daschner	1988	15		696	116	36	13	
Duszyńska	1988	16		8425	1097	93	7	
Fagon	1989	17	B	7371	567	49	17	
Gacouin	2009	18	B	3971	361	76	21	9
Garrouste-Orgas	1997	19	B	946	86	31	13	13
Georges	2000	20	B	2430	135	35	11	
Giard	2008	21	B	63067	7236	946	193	
Gruson-95-96	2000	22	B	11646	1004	231	67	40
Gruson-97-98	2000	22	B	12245	1029	161	54	20
Gruson-99-01	2003	23	B	9546	823	134	26	
Guérin	1997	24	B	2314	260	27	3	1
Hugonnet	2007	25	B	4493	936	209	55	
Hyllienmark	2007	26	B	1086	221	33	2	0
Jaillette	2011	27		6585	439	137	22	13
Kohlenberg	2010	28		1068472	779500	5811	1222	443
Kolpa	2018	29		12059	1270	183	16	
Lepelletier	2010	30	T	2093	161	34	34	5

Table S1: Studies reporting *S aureus* VAP incidence (continued)

Author	Year	Ref	Notes[a]	MV (days) [b]	Patients (n)	VAP (n)	*S aureus* VAP (n)	MRSA VAP (n)
**North Europe (continued)**								
Luyt	2005	31		10150	290	69	12	4
Magnason	2008	32		933	280	21	1	
Mahul	1992	33	B	2407	145	30	10	
Markowicz	2000	34	B	8704	744	162	74	40
Michel	2005	35	B	3942	299	41	12	1
Moine	2002	36	B	7411	764	89	19	14
Myny	2005	37		2310	385	89	27	7
Nguile-Makao	2010	38	B	20111	2873	434	89	
Nielsen	1992	39		919	242	23	5	
Nseir	2005	40		29784	1241	77	15	8
Papazian	1996	41	B	9376	586	97	20	
Reusser	1989	42		280	40	15	6	
Stéphan	2006	43	T	875	175	78	43	2
Timsit	1996	44	B	4644	387	56	18	
Trouillet	1998	45	B	8466	498	135	52	32
Vanhems	2011	46	B	17792	3387	367	137	
Verhamme	2007	47		22230	4000	298	56	7
Woske	2001	48	B	1545	103	49	29	1
Zahar	2009	49	B	10899	1233	208	51	

Table S1: Studies reporting *S aureus* VAP incidence (continued)

Author	Year	Ref	Notes[a]	MV (days) [b]	Patients (n)	VAP (n)	*S aureus* VAP (n)	MRSA VAP (n)
**Mediterranean**								
Alvarez-Lerma	1996	50	B	59744	6494	519	102	
Antonelli	1994	51		1476	124	41	10	
Apostolopoulou	2003	52		2205	175	56	9	3
Bouza	2003	53	C	399	356	28	5	
Cavalcanti	2006	54	B	950	190	62	18	
Cenderero	1999	55	B	799	123	19	9	
Chaari	2015	56		1435	175	48	6	
de_Latore	1995	57	B	816	80	12	3	
Ewig	1999	58	B	288	48	10	5	0
Hortal	2009	59	C	2692	231	106	38	25
Ibáñez	2000	60		228	30	6	3	
Jimenez	1989	61		431	77	18	2	
Kallel	2005	62		3301	241	77	15	2
Piazza	2006	63		2466	143	29	5	
Raineri	2010	64	B	3819	827	68	22	3
Raineri	2010	64	I, B	3019	822	44	11	4
Ramirez	2016	65	B	3080	440	71	8	
Rello'91	1991	66	B	2085	264	58	15	
Rello'92	1992	67	B, T		161	42	21	
Rello'96	1996	68	B	664	83	21	0	
Rello'03	2003	69		693	99	18	2	1
Rincón-Ferrari	2004	70	B	3100	310	72	27	
Ruiz-Santana	1987	71	B	7035	1005	180	12	8
Sofianou	2000	72		1584	198	67	13	13
Tamayo	2012	73	C	5799	1610	124	55	
Tejada-Artigas	2001	74	B	824	103	23	11	
Torres	1990	75	B	1288	322	78	2	
Urli	2002	76		3145	178	116	40	20
Valles	2007	77		720	60	40	9	0
Violan	1998	78	B	4082	314	82	26	1

Table S1: Studies reporting *S aureus* VAP incidence (continued)

Author	Year	Ref	Notes[a]	MV (days) [b]	Patients (n)	VAP (n)	*S aureus* VAP (n)	MRSA VAP (n)
**Asia**								
Apisarnthanarak	2007	79		2184	470	45	7	5
Apisarnthanarak	2007	79	I	2115	482	18	2	1
Apisarnthanarak	2007	79	I	4114	952	18	2	1
Mallick	2015	80		1707	301	61	4	4
Noor	2005	81		1500	250	70	11	7
Pawar	2003	82	C	919	952	25	4	
Salahuddin	2004	83		3140	333	43	3	
Salahuddin	2004	83	I	2905	344	19	3	
Tan	2016	84		5562	618	286	23	
Tao	2012	85		29041	16426	365	38	
Tao	2011	86		540536	391527	11224	922	
Thongpiyapoom	2004	87		6850	1076	74	1	
Xie	2011	88		30035	4155	868	92	41
**Middle East**								
Ertugrul	2006	89		970	100	28	12	11
Gursel	2010	90		920	92	59	13	12
Jacobs	1990	91		358	24	13	2	
Jehani-Sherafat	2015	92		6976	2584	55	26	
Kanafani	2003	93		1190	70	33	3	0
Leblebicioglu	2007	94		23520	3296	623	151	
Leblebicioglu	2013	95		2376	448	74	14	
Leblebicioglu	2013	95	I	28181	3864	474	51	
Memish	2000	96		3035	202	41	16	
Rezai	2017	97		5965	562	205	24	
Şimşek	2001	98	C, B	2708	1716	46	8	

Table S1: Studies reporting *S aureus* VAP incidence (continued)

Author	Year	Ref	Notes[a]	MV (days) [b]	Patients (n)	VAP (n)	*S aureus* VAP (n)	MRSA VAP (n)
**Central and South America**								
Berg	1995	99	S	888	253	62	10	
Guanche-Garcell	2013	100		114	67	6	0	
Guanche-Garcell	2013	100	I	2350	1008	36	1	
Guimaraes	2006	101		1946	278	106	7	7
Jaimes	2007	102		2592	270	60	10	13
Luna	2003	103	B	3245	427	63	19	
Moreno	2006	104		8593	2172	86	26	
Resende	2013	105		3024	126	33	5	
Rodrigues	2009	106		2330	233	64	11	
**North America**								
Baker	1996	107	B	5140	514	30	5	
Bochicchio	2004	108		7119	678	125	50	9
Braun	1986	109		396	66	15	6	
Cook_non-trauma	2010	110	B	11440	2080	70	14	
Cook_trauma	2010	110	T, B	3730	511	91	15	10
Craven-surgical	1988	111		1875	521	49	12	12
Craven-medical	1988	111		1108	277	47	9	
Ensminger	2006	112	C	2135	92	17	6	1
Evans	2010	113		3328	416	101	40	22
George	1998	114	B	1338	223	28	8	4
Heyland	1999	115	B	16224	1014	177	64	4
Ibrahim	2000	116		8845	1882	397	143	81
Ibrahim	2001	117		6248	880	132	36	26
Kasuya	2011	118		1292	111	31	19	12
Kollef' 93	1993	119		2049	277	43	9	
Kollef '95	1995	120		4808	314	87	17	8
Kollef '97	1997	121		2891	521	77	25	12

Table S1: Studies reporting *S aureus* VAP incidence (continued)

Author	Year	Ref	Notes[a]	MV (days) [b]	Patients (n)	VAP (n)	*S aureus* VAP (n)	MRSA VAP (n)
**North America (continued)**								
Kollef '97_pre	1997	122	C	882	353	42	5	4
Kollef '97_post	1997	122	C, I	784	327	23	4	3
Koss	2001	123		896	87	17	3	
Koss	2001	123	I	495	66	24	4	
Kunac	2014	124	B	3580	716	206	62	40
Lee	2013	125		192143		247	85	70
Lowy	1987	126			78	35	5	
Rello'02	2002	127		49032	9080	842	143	
Rodriguez	1991	128		3234	294	130	37	14
Salata	1987	129	B	688	51	21	2	
Shahin	2013	130		1602	267	29	6	
**Ungrouped**								
Boots	2008	131	B	4367	412	58	32	17
Cade	1993	132		392	98	35	13	10
Potgieter	1987	133		2300	250	51	23	6

Table S1 – footnotes

a. Abbreviations; B = Bronchoscopic sampling used toward VAP diagnosis, I = Intervention period; C = Cardiac ICU; T = Trauma ICU
b. MV = Mechanical ventilation
c. *S aureus* VAP including MRSA VAP in the total count.
d. The data from Blot [1] is derived from the EU-VAP multi-center prospective study from 27 ICU's across Europe and stratified by the following three age groups; middle-aged (45-64 yr; n = 670), old (65-74 yr; n = 549), and very old patients (≥ 75 yr; n= 516. Patients admitted for a trauma related diagnosis were not included in the data published by Blot [1] due to the strong relationship with younger age. The data for Magret [4] is also derived from the EU-VAP multi-center prospective study and is stratified by admission for a trauma related diagnosis or not. The complete data for all 2436 patients of the EU-VAP data set has also been published in aggregate [s134]

References

S1. Blot S, Koulenti D, Dimopoulos G, Martin C, Komnos A, Krueger WA, Spina G, Armaganidis A, Rello J, EU-VAP Study Investigators. Prevalence, risk factors, and mortality for ventilator-associated pneumonia in middle-aged, old, and very old critically ill patients. *Crit Care Med.* 2014;42(3):601-9.

S2. Chevret S, Hemmer M, Carlet J, et al. Incidence and risk factors of pneumonia acquired in intensive care units. Results from a multicenter prospective study on 996 patients. European Cooperative Group on Nosocomial Pneumonia. *Intensive Care Med*. 1993;19:256-264.

S3. Kollef MH, Chastre J, Fagon JY, François B, Niederman MS, Rello J, Torres A, Vincent JL, Wunderink RG, Go KW, Rehm C. Global prospective epidemiologic and surveillance study of ventilator-associated pneumonia due to *Pseudomonas aeruginosa*. *Crit Care Med* 2014;42(10):2178-87.

S4. Magret M, Amaya-Villar R, Garnacho J, Lisboa T, Diaz E, DeWaele J, Deja M, Manno E, Rello J, EU-VAP/CAP Study Group. Ventilator-associated pneumonia in trauma patients is associated with lower mortality: results from EU-VAP study. *J Trauma Acute Care Surg*. 2010;69(4):849-54.

S5. Rosenthal VD, Maki DG, Salomao R, Moreno CA, Mehta Y, Higuera F, Cuellar LE, Arikan OA, Abouqal R, Leblebicioglu H. Device-associated nosocomial infections in 55 intensive care units of 8 developing countries. *Ann Intern Med*. 2006;145(8):582-91.

S6. Rosenthal VD, Rodrigues C, Madani N, Mitrev Z, Ye G, Salomao R, Ulger F, Guanche-Garcell H, Kanj SS, Cuéllar LE, Higuera F. Effectiveness of a multidimensional approach for prevention of ventilator-associated pneumonia in adult intensive care units from 14 developing countries of four continents: Findings of the International Nosocomial Infection Control Consortium. *Crit Care Med*. 2012;40(12):3121-8.

S7. A'Court CH, Garrard CS, Crook D, et al. Microbiological lung surveillance in mechanically ventilated patients, using non-directed bronchial lavage and quantitative culture. *Q J Med.* 1993;86:635-48.

S8. Bekaert M, Timsit JF, Vansteelandt S, Depuydt P, Vésin A, Garrouste-Orgeas M, Decruyenaere J, Clec'h C, Azoulay E, Benoit D. AttribuTable Smortality of ventilator-associated pneumonia: a reappraisal using causal analysis. *Am J Respir Crit Care Med*. 2011;184(10):1133-9.

S9. Bercault N, Wolf M, Runge I, et al. Intrahospital transport of critically ill ventilated patients: a risk factor for ventilator-associated pneumonia--a matched cohort study. *Crit Care Med*. 2005;33:2471-8.

S10. Berrouane Y, Daudenthun I, Riegel B, Emery MN, Martin G, Krivosic R, Grandbastien B. Early onset pneumonia in neurosurgical intensive care unit patients. *J Hosp Infect.* 1998;40(4):275-80.

S11. Bornstain C, Azoulay E, De Lassence A, Cohen Y, Costa MA, Mourvillier B, Descorps-Declere A, Garrouste-Orgeas M, Thuong M, Schlemmer B, Timsit JF. Sedation, sucralfate, and antibiotic use are potential means for protection against early-onset ventilator-associated pneumonia. *Clin Infect Dis*. 2004;38(10):1401-8.

S12. Bregeon F, Papazian L, Visconti A, Gregoire R, Thirion X, Gouin F. Relationship of microbiologic diagnostic criteria to morbidity and mortality in patients with ventilator-associated pneumonia. *JAMA*. 1997,277: 655-662.

S13. Bronchard R, Albaladejo P, Brezac G, et al. Early onset pneumonia: risk factors and consequences in head trauma patients. *Anesthesiol* 2004;100:234-9.

S14. Chastre J, Trouillet JL, Vuagnat A, et al. Nosocomial pneumonia in patients with acute respiratory distress syndrome. *Am J Respir Crit Care Med* 1998;157:1165-72.

S15. Daschner F, Kappstein I, Schuster F, et al. Influence of disposable ('Conchapak') and reusable humidifying systems on the incidence of ventilation pneumonia. *J Hosp Infect*. 1988;11:161-168

S16. Duszyńska W, Rosenthal VD, Dragan B, Węgrzyn P, Mazur A, Wojtyra P, Tomala A, Kübler A. Ventilator-associated pneumonia monitoring according to the INICC project at one centre. *Anaesthesiology Intensive Ther.* 2015;47(1):34-9

S17. Fagon JY, Chastre J, Domart Y, Trouillet JL, Pierre J, Darne C, Gibert C. Nosocomial pneumonia in patients receiving continuous mechanical ventilation. Prospective analysis of 52 episodes with use of a protected specimen brush and quantitative culture techniques. *Am Rev Respir Dis*. 1989;139:877-884.

S18. Gacouin A, Barbarot N, Camus C, Salomon S, Isslame S, Marque S, Lavoué S, Donnio PY, Thomas R, Le Tulzo Y. Late-onset ventilator-associated pneumonia in nontrauma intensive care unit patients. *Anesthesia & Analgesia*. 2009;109(5):1584-90.

S19. Garrouste-Orgeas M, Chevret S, Arlet G, et al. Oropharyngeal or gastric colonization and nosocomial pneumonia in adult intensive care unit patients. A prospective study based on genomic DNA analysis. *Am J Respir Crit Care Med*. 1997;156:1647-56.

S20. Georges H, Leroy O, Guery B, Alfandari S, Beaucaire G. Predisposing factors for nosocomial pneumonia in patients receiving mechanical ventilation and requiring tracheotomy. *Chest*. 2000;118:767–774.

S21. Giard M, Lepape A, Allaouchiche B, Guerin C, Lehot JJ, Robert MO, Fournier G, Jacques D, Chassard D, Gueugniaud PY, Artru F. Early-and late-onset ventilator-associated pneumonia acquired in the intensive care unit: comparison of risk factors. J *Crit Care*. 2008;23(1):27-33.

S22. Gruson D, Hilbert G, Vargas F, Valentino R, Bebear C, Allery A, Bebear C, Gbikpi-benissan GE, Cardinaud JP. Rotation and restricted use of antibiotics in a medical intensive care unit: impact on the incidence of ventilator-associated pneumonia caused by antibiotic-resistant gram-negative bacteria. *Am J Respir Crit Care Med*. 2000;162(3):837-43.

S23. Gruson D, Hilbert G, Vargas F, et al. Strategy of antibiotic rotation: long-term effect on incidence and susceptibilities of Gram-negative bacilli responsible for ventilator-associated pneumonia. *Crit Care Med*. 2003;31:1908-1914.

S24. Guerin C, Girard R, Chemorin C, De Varax R, Fournier G. Facial mask noninvasive mechanical ventilation reduces the incidence of nosocomial pneumonia. *Intensive Care Med*. 1997;23(10):1024-32.

S25. Hugonnet S, Uçkay I, Pittet D. Staffing level: a determinant of late-onset ventilator-associated pneumonia. *Crit Care*. 2007;11(4), R80.

S26. Hyllienmark P, Gardlund B, Persson JO, Ekdahl K. Nosocomial pneumonia in the ICU: a prospective cohort study. *Scand J Infect Dis.* 2007;39:676-82.

S27. Jaillette E, Nseir S. Relationship between inhaled β2-agonists and ventilator-associated pneumonia: A cohort study. *Crit Care Med*. 2011;39(4):725-30.

S28. Kohlenberg A, Schwab F, Behnke M, Geffers C, Gastmeier P. Pneumonia associated with invasive and noninvasive ventilation: an analysis of the German nosocomial infection surveillance system database. *Intensive Care Med*. 2010;36(6):971-8.

S29. Kolpa M, Walaszek M, Gniadek A, Wolak Z, Dobros W. Incidence, Microbiological Profile and Risk Factors of Healthcare-Associated Infections in Intensive Care Units: A 10 Year Observation in a Provincial Hospital in Southern Poland. *Int. J. Environ. Res. Public Health* 2018;15, 112

S30. Lepelletier D, Roquilly A, Mahe PJ, Loutrel O, Champin P, Corvec S, Naux E, Pinaud M, Lejus C, Asehnoune K. Retrospective analysis of the risk factors and pathogens associated with early-onset ventilator-associated pneumonia in surgical-ICU head-trauma patients. *J Neurosurg Anesthesiol.* 2010;22(1):32-7.

S31. Luyt CE, Guerin V, Combes A, et al. Procalcitonin kinetics as a prognostic marker of ventilator-associated pneumonia. *Am J Respir Crit Care Med*. 2005;171:48-53.

S32. Magnason S, Kristinsson KG, Stefansson T, Erlendsdottir H, Jonsdottir K, Kristjansson M, Gudmundsson S. Risk factors and outcome in ICU-acquired infections. *Acta Anaesthesiologica Scand.* 2008;52:1238-1245.

S33. Mahul P, Auboyer C, Jospe R, Ros A, Guerin C, el Khouri Z, Galliez M, Dumont A, Gaudin O. Prevention of nosocomial pneumonia in intubated patients respective role of mechanical subglottic secretions drainage and stress ulcer prophylaxis. *Intensive Care Med*. 1992;18:20-25.

S34. Markowicz P, Wolff M, Djedaini K, Cohen Y, Chastre J, Delclaux C. Multicenter prospective study of ventilator-associated pneumonia during acute respiratory distress syndrome. Incidence, prognosis, and risk factors. ARDS Study Group. *Am J Respir Crit Care Med*. 2000;161:1942-8.

S35. Michel F, Franceschini B, Berger P, Arnal JM, Gainnier M, Sainty JM, Papazian L. Early antibiotic treatment for BAL-confirmed ventilator-associated pneumonia: a role for routine endotracheal aspirate cultures. *Chest*. 2005;127(2):589-97.

S36. Moine P, Timsit JF, De Lassence A, et al. Mortality associated with late-onset pneumonia in the intensive care unit: results of a multi-center cohort study. *Intensive Care Med*. 2002;28:154-63.

S37. Myny D, Depuydt P, Colardyn F, Blot S. Ventilator-associated pneumonia in a tertiary care ICU analysis of risk factors for acquisition and mortality. *Acta Clin Belg.* 2005;60:114-121.

S38. Nguile-Makao M, Zahar JR, Français A, Tabah A, Garrouste-Orgeas M, Allaouchiche B, Goldgran-Toledano D, Azoulay E, Adrie C, Jamali S, Clec'h C. AttribuTable Smortality of ventilator-associated pneumonia: respective impact of main characteristics at ICU admission and VAP onset using conditional logistic regression and multi-state models. *Intens Care Med.* 2010;36(5):781-9.

S39. Nielsen SL, Roder B, Magnussen P, et al. Nosocomial pneumonia in an intensive care unit in a Danish university hospital: incidence, mortality and etiology. *Scand J Infect Dis*. 1992;24:65-70.

S40. Nseir S, Di Pompeo C, Soubrier S, Cavestri B, Jozefowicz E, Saulnier F, Durocher A. Impact of ventilator-associated pneumonia on outcome in patients with COPD. *Chest*. 2005;128(3):1650-6.

S41. Papazian L, Bregeon F, Thirion X, et al. Effect of ventilator-associated pneumonia on mortality and morbidity. *Am J Respir Crit Care Med*. 1996;154:91-7.

S42. Reusser P, Zimmerli W, Scheidegger D, Marbet GA, Buser M, Gyr K. Role of gastric colonization in nosocomial infections and endotoxemia: a prospective study in neurosurgical patients on mechanical ventilation. *J Infect Dis*. 1989;160:414-421.

S43. Stéphan F, Mabrouk N, Decailliot F, Delclaux C, Legrand P. Ventilator-associated pneumonia leading to acute lung injury after trauma: importance of *Haemophilus influenzae*. *Anesthesiol*. 2006;104:235-41.

S44. Timsit JF, Chevret S, Valcke J, et al. Mortality of nosocomial pneumonia in ventilated patients: influence of diagnostic tools. *Am J Respir Crit Care Med*. 1996;154:116-23.

S45. Trouillet JL, Chastre J, Vuagnat A, Joly-Guillou ML, Combaux D, Dombret MC, Gibert C. Ventilator-associated pneumonia caused by potentially drug-resistant bacteria. *Am J Respir Crit Care Med*. 1998;157(2):531-9.

S46. Vanhems P, Bénet T, Voirin N, Januel JM, Lepape A, Allaouchiche B, Argaud L, Chassard D, Guérin C. Early-onset ventilator-associated pneumonia incidence in intensive care units: a surveillance-based study. *BMC Infect Dis*. 2011;11(1):236.

S47. Verhamme KM, De Coster W, De Roo L, De Beenhouwer H, Nollet G, Verbeke J, Demeyer I, Jordens P. Pathogens in early-onset and late-onset intensive care unit–acquired pneumonia. *Infect Control Hosp Epidemiol*. 2007;28(04):389-97.

S48. Woske HJ, Röding T, Schulz I, Lode H. Ventilator-associated pneumonia in a surgical intensive care unit Epidemiology, etiology and comparison of three bronchoscopic methods for microbiological specimen sampling. *Crit Care*. 2001;5:167–173.

S49. Zahar JR, Nguile-Makao M, Français A, Schwebel C, Garrouste-Orgeas M, Goldgran-Toledano D, Azoulay E, Thuong M, Jamali S, Cohen Y, De Lassence A. Predicting the risk of documented ventilator-associated pneumonia for benchmarking: construction and validation of a score. *Crit Care Med*. 2009;37(9):2545-51.

S50. Alvarez-Lerma F, ICU-acquired Pneumonia Study Group. Modification of empiric antibiotic treatment in patients with pneumonia acquired in the intensive care unit. *Intensive Care Med.* 1996;22(5):387-94.

S51. Antonelli M, Moro ML, Capelli O, et al. Risk factors for early onset pneumonia in trauma patients. *Chest*. 1994;105:224-228.

S52. Apostolopoulou E, Bakakos P, Katostaras T, et al. Incidence and risk factors for ventilator-associated pneumonia in 4 multidisciplinary intensive care units in Athens, Greece. *Respir Care.* 2003;48:681-8.

S53. Bouza E, Pérez A, Muñoz P, Perez MJ, Rincón C, Sánchez C, Martín-Rabadán P, Riesgo M, Cardiovascular Infection Study Group. Ventilator-associated pneumonia after heart surgery: a prospective analysis and the value of surveillance. *Crit Care Med*. 2003;31(7):1964-70.

S54. Cavalcanti M, Ferrer M, Ferrer R, et al. Risk and prognostic factors of ventilator-associated pneumonia in trauma patients. *Crit Care Med*. 2006;34:1067-1072.

S55. Cardenosa Cendrero JA, Sole-Violan J, Bordes Benitez A, et al. Role of different routes of tracheal colonization in the development of pneumonia in patients receiving mechanical ventilation. *Chest*. 1999;116:462-470.

S56. Chaari A, El Habib M, Ghdhoun H, Algia NB, Chtara K, Hamida CB, Chelly H, Bahloul M, Bouaziz M. Does low-dose hydrocortisone therapy prevent ventilator-associated pneumonia in trauma patients? *Am J Therapeut.* 2015;22(1):22-8.

S57. de Latorre FJ, Pont T, Ferrer A, et al. Pattern of tracheal colonization during echanical ventilation. *Am J Respir Crit Care Med*. 1995;152:1028-1033.

S58. Ewig S, Torres A, El-Ebiary M, et al. Bacterial colonization patterns in mechanically ventilated patients with traumatic and medical head injury. Incidence, risk factors, and association with ventilator-associated pneumonia. *Am J Respir Crit Care Med*. 1999;159:188-198.

S59. Hortal J, Giannella M, Pérez MJ, Barrio JM, Desco M, Bouza E, Muñoz P. Incidence and risk factors for ventilator-associated pneumonia after major heart surgery. *Intensive Care Med.* 2009;35(9):1518-25.

S60. Ibáñez J, Peñafiel A, Marsé P, Jordá R, Raurich JM, Mata F. Incidence of gastroesophageal reflux and aspiration in mechanically ventilated patients using small-bore nasogastric tubes. *JPEN J Parenter Enteral Nutr*. 2000;24(2):103-6.

S61. Jimenez P, Torres A, Rodriguez-Roisin R, et al. Incidence and etiology of pneumonia acquired during mechanical ventilation. *Crit Care Med*. 1989;17:882-5.

S62. Kallel H, Chelly H, Bahloul M, Ksibi H, Dammak H, Chaari A, Hamida CB, Rekik N, Bouaziz M. The effect of ventilator-associated pneumonia on the prognosis of head trauma patients. *J Trauma Acute Care Surg*. 2005;59(3):705-10.

S63. Piazza O, Iasiello A, PapaIanni C, De Robertis E, Servillo G, Rossano F, Tufano R. Incidence of antimicrobial-resistant ventilator associated pneumonia: an eighteen-month survey. *Panminerva medica.* 2005;47(4):265-7.

S64. Raineri E, Crema L, Dal Zoppo S, Acquarolo A, Pan A, Carnevale G, Albertario F, Candiani A. Rotation of antimicrobial therapy in the intensive care unit: impact on incidence of ventilator-associated pneumonia caused by antibiotic-resistant Gram-negative bacteria. *Eur J Clin Microbiol Infect Dis*. 2010;29(8):1015-24.

S65. Ramirez P, Lopez-Ferraz C, Gordon M, Gimeno A, Villarreal E, Ruiz J, Menendez R, Torres A. From starting mechanical ventilation to ventilator-associated pneumonia, choosing the right moment to start antibiotic treatment. *Crit Care* 2016;20(1):169.

S66. Rello J, Quintana E, Ausina V, Castella J, Luquin M, Net A, Prats G. Incidence, etiology, and outcome of nosocomial pneumonia in mechanically ventilated patients. *Chest*. 1991;100(2):439-44.

S67. Rello J, Ausina V, Castella J, Net A, Prats G. Nosocomial respiratory tract infections in multiple trauma patients: influence of level of consciousness with implications for therapy. *Chest*. 1992;102(2):525-9.

S68. Rello J, Sonora R, Jubert P, Artigas A, Rué M, Vallés J. Pneumonia in intubated patients: role of respiratory airway care. *Am J Respir Crit Care Med*. 1996;154(1):111-5.

S69. Rello J, Lorente C, Diaz E, et al. Incidence, etiology, and outcome of nosocomial pneumonia in ICU patients requiring percutaneous tracheotomy for mechanical ventilation. *Chest*. 2003;124:2239-2243.

S70. Rincón-Ferrari MD, Flores-Cordero JM, Leal-Noval SR, Murillo-Cabezas F, Cayuelas A, Muñoz-Sánchez MA, Sánchez-Olmedo JI. Impact of ventilator-associated

pneumonia in patients with severe head injury. *J Trauma Acute Care Surg.* 2004;57(6):1234-40.

S71. Ruiz-Santana S, Garcia Jimenez A, Esteban A, et al. ICU pneumonias: a multi-institutional study. *Crit Care Med*. 1987;15:930-932.

S72. Sofianou DC, Constandinidis TC, Yannacou M, et al. Analysis of risk factors for ventilator-associated pneumonia in a multidisciplinary intensive care unit. *Eur J Clin Microbiol Infect Dis.* 2000;19:460-3.

S73. Tamayo E, Álvarez FJ, Martínez-Rafael B, Bustamante J, Bermejo-Martin JF, Fierro I, Eiros JM, Castrodeza J, Heredia M, Gómez-Herreras JI, Valladolid Sepsis Study Group. Ventilator-associated pneumonia is an important risk factor for mortality after major cardiac surgery. *J Crit Care*. 2012;27(1):18-25.

S74. Tejada Artigas A, Bello Dronda S, Chacon Valles E, et al. Risk factors for nosocomial pneumonia in critically ill trauma patients. *Crit Care Med*. 2001;29:304-9.

S75. Torres A, Aznar R, Gatell JM, et al. Incidence, risk, and prognosis factors of nosocomial pneumonia in mechanically ventilated patients. *Am Rev Respir Dis*. 1990;142:523-8.

S76. Urli T, Perone G, Acquarolo A, Zappa S, Antonini B, Candiani A. Surveillance of infections acquired in intensive care: usefulness in clinical practice. *J Hosp Infect.* 2002;52(2):130-5.

S77. Valles J, Pobo A, Garcia-Esquirol O, Mariscal D, Real J, Fernandez R. Excess ICU mortality attribuTable Sto ventilator-associated pneumonia: the role of early vs late onset. *Intensive Care Med*. 2007;33(8):1363.

S78. Violan JS, Sanchez-Ramirez C, Mujica AP, Cendrero JC, Fernandez JA, de Castro FR. Impact of nosocomial pneumonia on the outcome of mechanically-ventilated patients. *Crit Care* (Lond). 1998;2:19-23.

S79. Apisarnthanarak A, Pinitchai U, Thongphubeth K, Yuekyen C, Warren DK, Zack JE, Warachan B, Fraser VJ. Effectiveness of an educational program to reduce ventilator-associated pneumonia in a tertiary care center in Thailand: a 4-year study. *Clin Infect Dis*. 2007;45(6):704-11.

S80. Mallick UK, Faruq MO, Ahsan AA, Fatema K, Ahmed F, Asaduzzaman M, Islam M, Sultana A. Spectrum of Early Onset and Late Onset Ventilator Associated Pneumonia (VAP) in a Tertiary Care Hospital of Bangladesh: A Prospective Cohort Study. *Bangladesh Crit Care J.* 2015;3(1):9-13.

S81. Noor A, Hussain SF. Risk factors associated with development of ventilator associated pneumonia. *J Coll Physicians Surg Pak.* 2005;15:92–95.

S82. Pawar M, Mehta Y, Khurana P, Chaudhary A, Kulkarni V, Trehan N. Ventilator-associated pneumonia: incidence, risk factors, outcome, and microbiology. *J Cardiothoracic Vascular Anesthesia*. 2003;17(1):22-8.

S83. Salahuddin N, Zafar A, Sukhyani L, Rahim S, Noor MF, Hussain K, Siddiqui S, Islam M, Husain SJ. Reducing ventilator-associated pneumonia rates through a staff education programme. *J Hosp Infect.* 2004;57(3):223-7.

S84. Tan X, Zhu S, Yan D, Chen W, Chen R, Zou J, Yan J, Zhang X, Farmakiotis D, Mylonakis E. *Candida* spp. airway colonization: A potential risk factor for *Acinetobacter baumannii* ventilator-associated pneumonia. *Med Mycol*. 2016:myw009.

S85. Tao L, Hu B, Rosenthal VD, Gao X, He L. Device-associated infection rates in 398 intensive care units in Shanghai, China: International Nosocomial Infection Control Consortium (INICC) findings. *Int J Infect Dis.* 2011;15(11):e774-80.

S86. Tao L, Hu B, Rosenthal VD, Zhang Y, Gao X, He L. Impact of a multidimensional approach on ventilator-associated pneumonia rates in a hospital of Shanghai: findings of the International Nosocomial Infection Control Consortium. *J Crit Care.* 2012;27(5):440-6.

S87. Thongpiyapoom S, Narong MN, Suwalak N, Jamulitrat S, Intaraksa P, Boonrat J, Kasatpibal N, Unahalekhaka A. Device-associated infections and patterns of antimicrobial resistance in a medical-surgical intensive care unit in a university hospital in Thailand. *J Med Assoc Thailand.* 2004;87(7):819-24.

S88. Xie DS, Xiong W, Lai RP, Liu L, Gan XM, Wang XH, Wang M, Lou YX, Fu XY, Wang HF, Xiang H. Ventilator-associated pneumonia in intensive care units in Hubei Province, China: a multicentre prospective cohort survey. *J Hosp Infect.* 2011;78(4):284-8.

S89. Ertugrul BM, Yildirim A, Ay P, Oncu S, Cagatay A, Cakar N, Ertekin C, Ozsut H, Eraksoy H, Calangu S. Ventilator-associated pneumonia in surgical emergency intensive care unit. *Saudi Med J.* 2006;27(1):52-7.

S90. Gursel G, Aydogdu M, Nadir Ozis T, Tasyurek S. Comparison of the value of initial and serial endotracheal aspirate surveillance cultures in predicting the causative pathogen of ventilator-associated pneumonia. *Scand J Infect Dis.* 2010;42:341-346.

S91. Jacobs S, Chang RW, Lee B, Bartlett FW. Continuous enteral feeding: a major cause of pneumonia among ventilated intensive care unit patients. *JPEN J Parenter Enteral Nutr* 1990; 14:353-356.

S92. Jahani-Sherafat S, Razaghi M, Rosenthal VD, Tajeddin E, Seyedjavadi S, Rashidan M, Alebouyeh M, Rostampour M, Haghi A, Sayarbayat M, Farazmandian S. Device-associated infection rates and bacterial resistance in six academic teaching hospitals of Iran: findings from the International Nocosomial Infection Control Consortium (INICC). *J Infect Pub Health.* 2015;8(6):553-61.

S93. Kanafani ZA, Kara L, Hayek S, et al. Ventilator-associated pneumonia at a tertiary-care center in a developing country: incidence, microbiology, and susceptibility patterns of isolated microorganisms. *Infect Control Hosp Epidemiol*. 2003;24:864-9.

S94. Leblebicioglu H, Rosenthal VD, Arıkan ÖA, Özgültekin A, Yalcin AN, Koksal I, Usluer G, Sardan YC, Ulusoy S. Device-associated hospital-acquired infection rates in Turkish intensive care units. Findings of the International Nosocomial Infection Control Consortium (INICC). *J Hosp Infect.* 2007;65(3):251-7.

S95. Leblebicioglu H, Yalcin AN, Rosenthal VD, Koksal I, Sirmatel F, Unal S, Turgut H, Ozdemir D, Ersoz G, Uzun C, Ulusoy S. Effectiveness of a multidimensional approach for prevention of ventilator-associated pneumonia in 11 adult intensive care units from 10 cities of Turkey: findings of the International Nosocomial Infection Control Consortium (INICC). *Infect*. 2013;41(2):447-56.

S96. Memish ZA, Cunningham G, Oni GA, et al. The incidence and risk factors of ventilator-associated pneumonia in a Riyadh hospital. *Infect Control Hosp Epidemiol.* 2000;21:271-3.

S97. Rezai MS, Bagheri–Nesami M, Nikkhah A, Bayg AH. Incidence, risk factors, and outcome of ventilator-associated Pneumonia in 18 hospitals of Iran. Running title: ventilator-associated pneumonia in Iran. *Int J Adv Biotech Res.* 2016;7(3):936-46.

S98. Şimşek S, Yurtseven N, Gercekogalu H, Izgi F, Sohtorik Ü, Canik S, Özler A. Ventilator-associated pneumonias in a cardiothoracic surgery centre postoperative intensive care unit. *J Hosp Infect.* 2001;47(4):321-4.

S99. Berg DE, Hershow RC, Ramirez CA, Weinstein RA. Control of nosocomial infections in an intensive care unit in Guatemala City. *Clin Infect Dis*. 1995;21:588-593.

S100. Guanche-Garcell H, Morales-Perez C, Rosenthal VD. Effectiveness of a multidimensional approach for the prevention of ventilator-associated pneumonia in an adult intensive care unit in Cuba: findings of the International Nosocomial Infection Control Consortium (INICC). *J Infect Public Health.* 2013;6:98-107.

S101. Guimaraes MM, Rocco JR. Prevalence of ventilator-associated pneumonia in a university hospital and prognosis for the patients affected. *J Bras Pneumol.* 2006;32:339–346.

S102. Jaimes F, De La Rosa G, Gómez E, Múnera P, Ramírez J, Castrillón S. Incidence and risk factors for ventilator-associated pneumonia in a developing country Where is the difference? *Respir Med.* 2007;101:762–767.

S103. Luna CM, Blanzaco D, Niederman MS, et al. Resolution of ventilator-associated pneumonia: prospective evaluation of the clinical pulmonary infection score as an early clinical predictor of outcome. *Crit Care Med*. 2003;31:676-682.

S104. Moreno CA, Rosenthal VD, Olarte N, Gomez WV, Sussmann O, Agudelo JG, Rojas C, Osorio L, Linares C, Valderrama A, Mercado PG. Device-associated infection rate and mortality in intensive care units of 9 Colombian hospitals: findings of the International Nosocomial Infection Control Consortium. *Infect Control.* 2006;27(04):349-56.

S105. Resende MM, Monteiro SG, Callegari B, Figueiredo PM, Monteiro CR, Monteiro-Neto V. Epidemiology and outcomes of ventilator-associated pneumonia in northern Brazil: an analytical descriptive prospective cohort study. *BMC Infect Dis*. 2013;13(1):119.

S106. Rodrigues PM, Neto C, Santos LR, Knibel MF. Ventilator-associated pneumonia: epidemiology and impact on the clinical evolution of ICU patients. *J Brasileiro de Pneumologia.* 2009;35(11):1084-91.

S107. Baker AM, Meredith JW, Haponik EF. Pneumonia in intubated trauma patients. Microbiology and outcomes. *Am J Respir Crit Care Med*. 1996;153:343-9.

S108. Bochicchio GV, Joshi M, Bochicchio K, et al. A time-dependent analysis of intensive care unit pneumonia in trauma patients. *J Trauma.* 2004;56:296-301.

S109. Braun SR, Levin AB, Clark KL: Role of corticosteroids in the development of pneumonia in mechanically ventilated head-trauma victims. *Crit Care Med* 1986; 14:198-201.

S110. Cook A, Norwood S, Berne J. Ventilator-associated pneumonia is more common and of less consequence in trauma patients compared with other critically ill patients. *J Trauma Acute Care Surg.* 2010;69(5):1083-91.

S111. Craven DE, Kunches LM, Lichtenberg DA, et al. Nosocomial infection and fatality in medical and surgical intensive care unit patients. *Arch Intern Med.* 1988;148:1161-8.

S112. Ensminger SA, Wright RS, Baddour LM, Afess B. Suspected ventilator-associated pneumonia in cardiac patients admitted to the coronary care unit. *Mayo Clin. Proc.* 2006;81:32–35.

S113. Evans HL, Zonies DH, Warner KJ, Bulger EM, Sharar SR, Maier RV, Cuschieri J. Timing of intubation and ventilator-associated pneumonia following injury. *Arch Surg*. 2010;145(11):1041-6.

S114. George DL, Falk PS, Wunderink RG, Leeper Jr KV, Meduri GU, Steere EL, Glen Mayhall C. Epidemiology of ventilator-acquired pneumonia based on protected bronchoscopic sampling. *Am J Respir Crit Care Med*. 1998;158:1839-1847.

S115. Heyland DK, Cook DJ, Griffith L, Keenan SP, Brun-Buisson C. The attribuTable Smorbidity and mortality of ventilator-associated pneumonia in the critically ill patient. The Canadian Critical Trials Group. *Am J Respir Crit Care Med*. 1999;159:1249-1256.

S116. Ibrahim EH, Ward S, Sherman G, Kollef MH. A comparative analysis of patients with early-onset vs late-onset nosocomial pneumonia in the ICU setting. *Chest*. 2000;117:1434-1442.

S117. Ibrahim EH, Hill C, Fraser V, Kollef MH. The Occurrence Of Ventilator-associated Pneumonia (vap) In A Community Hospital: Risk Factors And Clinical Outcomes. Chest. 2000;118(4):555-561.

S118. Kasuya Y, Hargett JL, Lenhardt R, Heine MF, Doufas AG, Remmel KS, Ramirez JA, Akça O. Ventilator-associated pneumonia in critically ill stroke patients: frequency, risk factors, and outcomes. *J Crit Care.* 2011;26(3):273-9.

S119. Kollef MH. Ventilator-associated pneumonia. A multivariate analysis. *JAMA.* 1993;270:1965-70.

S120. Kollef MH, Silver P, Murphy DM, Trovillion E. The effect of late-onset ventilator-associated pneumonia in determining patient mortality. *Chest*. 1995;108(6):1655-62.

S121. Kollef MH, Vlasnik JO, Sharpless L, Pasque C, Murphy D, Fraser V. Scheduled change of antibiotic classes: a strategy to decrease the incidence of ventilator-associated pneumonia. *Am J Respir Crit Care Med*. 1997;156(4):1040-8.

S122. Kollef MH, Shapiro SD, Von Harz B, Prentice D, John RS, Silver P, Trovillion E. Patient transport from intensive care increases the risk of developing ventilator-associated pneumonia. *Chest*. 1997;112(3):765-73.

S123. Koss WG, Khalili TM, Lemus JF, Chelly MM. Nosocomial pneumonia is not prevented by protective contact isolation in the surgical intensive care unit. *Am Surg*. 2001;67(12):1140.

S124. Kunac A, Sifri ZC, Mohr AM, Horng H, Lavery RF, Livingston DH. Bacteremia and Ventilator-Associated Pneumonia: A Marker for Contemporaneous Extra-Pulmonic Infection. *Surg Infect.* 2014;15:77-83.

S125. Lee MS, Walker V, Chen LF, Sexton DJ, Anderson DJ. The epidemiology of ventilator-associated pneumonia in a network of community hospitals: a prospective multicenter study. *Infect Control Hosp Epidemiol*. 2013;34(7):657-62.

S126. Lowy FD, Carlisle PS, Adams A, Feiner C. The incidence of nosocomial pneumonia following urgent endotracheal intubation. *Infect Control Hosp Epidemiol*. 1987;8(6):245-8.

S127. Rello J, Ollendorf DA, Oster G, Vera-Llonch M, Bellm L, Redman R, Kollef MH. Epidemiology and outcomes of ventilator-associated pneumonia in a large US database. *Chest*. 2002;122(6):2115-21.

S128. Rodriguez JL, Gibbons KJ, Bitzer LG, et al. Pneumonia: incidence, risk factors, and outcome in injured patients. *J Trauma.* 1991;31:907-12.

S129. Salata RA, Lederman MM, Shlaes DM, Jacobs MR, Eckstein E, Tweardy D, Toossi Z, Chmielewski R, Marino J, King CH. Diagnosis of nosocomial pneumonia in intubated, intensive care unit patients. *Am Rev Respir Dis*. 1987;135:426-432.

S130. Shahin J, Bielinski M, Guichon C, Flemming C, Kristof AS. Suspected ventilator-associated respiratory infection in severely ill patients: a prospective observational study. *Crit Care*. 2013;22;17(5):R251.

S131. Boots RJ, Phillips GE, George N, Faoagali JL. Surveillance culture utility and safety using low-volume blind bronchoalveolar lavage in the diagnosis of ventilator-associated pneumonia. *Respirology*. 2008;13:87-96.

S132. Cade JF, McOwat E, Siganporia R, Keighley C, Presneill J, Sinickas V. Uncertain relevance of gastric colonization in the seriously ill. *Intensive Care Med*. 1992;18:210-7.

S133. Potgieter PD, Linton DM, Oliver S, Forder AA. Nosocomial infections in a respiratory intensive care unit. *Crit Care Med*. 1987;15:495-498.

S134. Koulenti D, Lisboa T, Brun-Buisson C, Krueger W, Macor A, Sole-Violan J, Diaz E, Topeli A, DeWaele J, Carneiro A, Martin-Loeches I. Spectrum of practice in the diagnosis of nosocomial pneumonia in patients requiring mechanical ventilation in European intensive care units. *Crit Care Med*. 2009;37(8):2360-9.

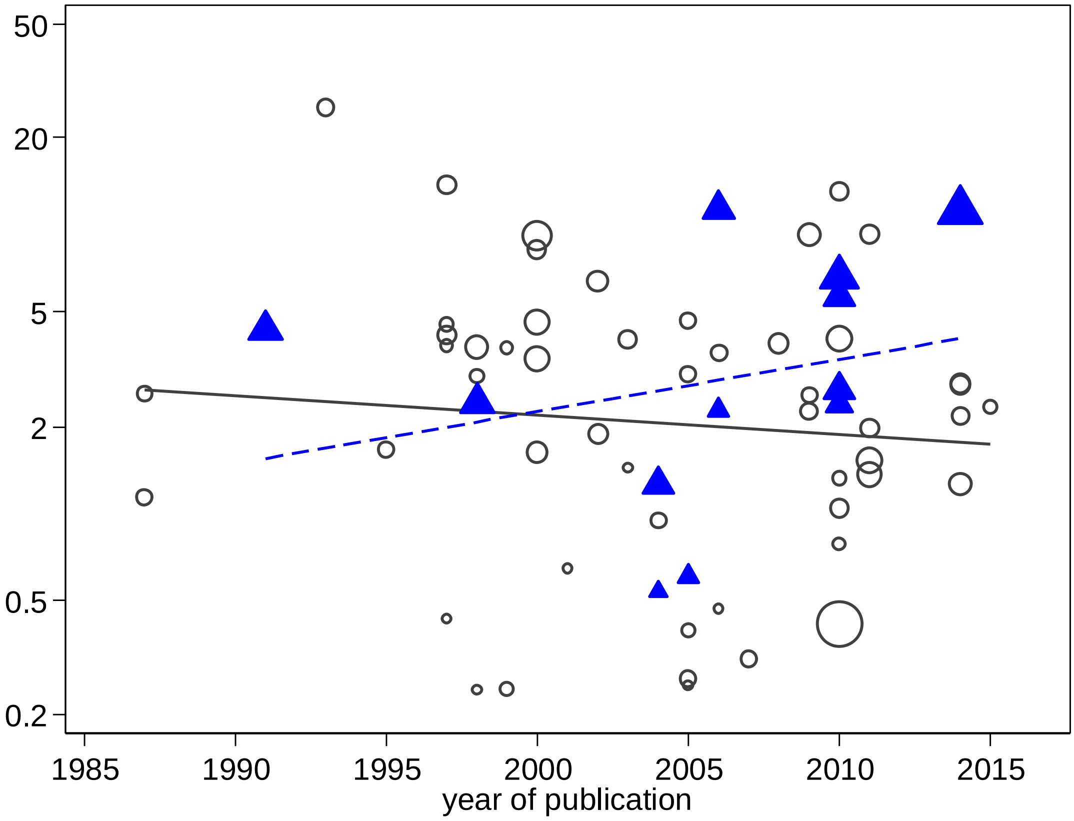

Fig S1. Scatter plot and linear regression of MRSA-VAP incidence (per 1000 MV days) versus year of study publication for studies reporting from trauma ICU's (triangles, broken line) versus non-trauma ICU's (circles, unbroken line). Note logarithmic scale of incidence. The slopes of the linear regression lines are significantly different (test for interaction p <0.001; Poisson regression).

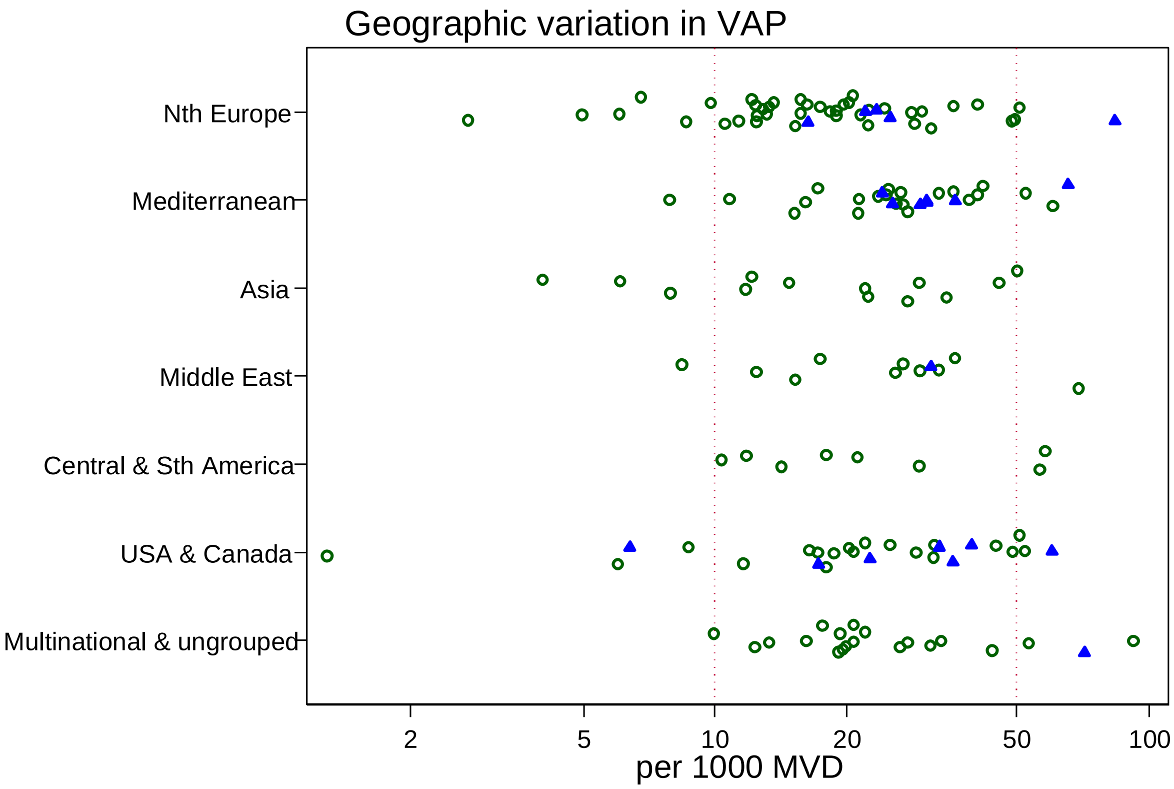

Fig S2. A scatter plot of VAP overall incidence per 1000 MVD among published studies in seven geographic regions with rates from trauma ICU's (closed symbols) versus other ICU's (open symbols). Note logit scale of incidence. The vertical lines at incidences 10 & 50 per 1000 MVD are for reference.
